# 2024 Update of the Asia‐Pacific League of Associations for Rheumatology (APLAR) Recommendations for Rheumatoid Arthritis Management

**DOI:** 10.1111/1756-185x.70752

**Published:** 2026-06-26

**Authors:** Kunihiro Yamaoka, Zhanguo Li, Khin Thin Zar Myo Aung, Paul Bird, Manisha Bhochhibhoya, Minhaj Rahim Choudhury, Debashish Danda, Tsolmon Darisuren, Rudy Hidayat, Nguyen Van Hung, Yuko Kaneko, Wanruchada Katchamart, Manjari Lahiri, Chin Teck Ng, Babur Salim, Keshav Raj Sigdel, Shereen Ch'ng Suyin, Tsutomu Takeuchi, Galymzhan Togizbayev, Evan Vista, Sandini Wijeweera, Chuanhui Xu, Chak Sing Lau

**Affiliations:** ^1^ Department of Rheumatology Kitasato University School of Medicine Sagamihara Japan; ^2^ Department of Rheumatology and Immunology Peking University People's Hospital Beijing China; ^3^ Department of Rheumatology Mandalay General Hospital Mandalay Myanmar; ^4^ School of Clinical Medicine University of New South Wales Sydney New South Wales Australia; ^5^ Department of Rheumatology National Center for Rheumatic Diseases (NCRD) Kathmandu Nepal; ^6^ Department of Rheumatology Bangladesh Medical University Dhaka Bangladesh; ^7^ Department of Clinical Immunology and Rheumatology Christian Medical College Vellore India; ^8^ Department of Rheumatology Mongolian National University of Medical Sciences Ulaanbaatar Mongolia; ^9^ Division of Rheumatology, Department of Internal Medicine Faculty of Medicine Universitas Indonesia, Cipto Mangunkusumo General Hospital Jakarta Indonesia; ^10^ Centre for Rheumatology, Bach Mai Hospital ‐ Department of Internal Medicine Hanoi Medical University Hanoi Vietnam; ^11^ Division of Rheumatology, Department of Internal Medicine Keio University School of Medicine Tokyo Japan; ^12^ Division of Rheumatology, Department of Medicine, Faculty of Medicine Siriraj Hospital Mahidol University Bangkok Thailand; ^13^ Department of Medicine Yong Loo Lin School of Medicine, National University of Singapore Singapore Singapore; ^14^ Department of Rheumatology and Immunology Singapore General Hospital Singapore Singapore; ^15^ Department of Rheumatology Foundation University School of Health Sciences, Fauji Foundation Hospital Rawalpindi Pakistan; ^16^ Department of Internal Medicine Patan Academy of Health Sciences Lalitpur Nepal; ^17^ Rheumatology Unit Selayang Hospital Selangor Malaysia; ^18^ Saitama Medical University, Saitama and Keio University Tokyo Japan; ^19^ JSC Research Institute of Cardiology and Internal Disease Almaty Kazakhstan; ^20^ Rheumatology, Allergy & Immunology Center St. Luke's Medical Center Quezon City Philippines; ^21^ Department of Rheumatology District General Hospital Negombo Sri Lanka; ^22^ Department of Rheumatology, Allergy and Immunology Tan Tock Seng Hospital Singapore Singapore; ^23^ Division of Rheumatology and Clinical Immunology, Department of Medicine, LKS Faculty of Medicine The University of Hong Kong Hong Kong Hong Kong

**Keywords:** biological DMARD, disease activity, guideline, rheumatoid arthritis, targeted synthetic DMARDs: conventional synthetic DMARDs

## Abstract

**Aim:**

To update the Asia‐Pacific League of Associations for Rheumatology (APLAR) treatment recommendations for rheumatoid arthritis (RA) by incorporating new evidence and reassessing the validity of previous guidance:

**Methods:**

A literature search for relevant publications from January 2018 to August 2023 was conducted, supplemented by earlier studies where relevant. Recommendations were formulated based on this evidence and refined through a modified‐Delphi process during a series of online meetings. The quality of evidence and strength of recommendations were assessed using the Grading of Recommendations, Assessment, Development and Evaluations (GRADE) approach.

**Results:**

This update comprises 14 statements that achieved consensus during the meetings. Topics covered include measurement of disease activity and treatment response, the use of conventional synthetic disease‐modifying antirheumatic drugs (csDMARDs), the use of targeted therapies, vaccination and screening for infections and the use of DMARDs during pregnancy. The recommendations address the selection of disease activity scores, initiation and intensification of therapy using csDMARDs and targeted therapies, and management of RA in patients at risk of reactivation of infectious diseases. These recommendations are discussed in the context of the clinical landscape encountered in the Asia‐Pacific region.

**Conclusion:**

These updated recommendations complement previous APLAR guidelines and aim to support best practice management of RA across the Asia‐Pacific region. They provide a practical framework for national rheumatology associations to develop local guidelines adapted to their specific needs and clinical practice.

## 1 Introduction

Rheumatoid arthritis (RA) is a chronic inflammatory autoimmune disease that is characterized by inflammation, pain, stiffness, and progressive joint degradation, which cause substantial morbidity and mortality if inadequately treated [1]. RA is also associated with lost productivity and consequent socioeconomic burdens as well as diminished quality of life [2]. Although the prevalence of RA in Asia (~0.3%) is lower than that reported in North America or Europe (0.5%–1%) [1, 3], the absolute prevalence of RA is high, and the incidence of RA and the associated burden are increasing [2].

Disease‐modifying antirheumatic drugs (DMARDs) are the mainstay of RA treatment [1]. Timely treatment with DMARDs effectively alters the clinical course, with slowing or cessation of joint damage [1, 4]. Frequently used conventional synthetic DMARDs (csDMARDs) include methotrexate (MTX), leflunomide, sulfasalazine (SSZ), and hydroxychloroquine (HCQ) [1], and other csDMARDs may be available in different countries/regions. Biologic DMARDs (bDMARDs) with diverse mechanisms of action are available, including tumor necrosis factor (TNF) inhibitors, interleukin‐6 (IL‐6) inhibitors, modulators of T‐cell costimulation, and anti‐CD20 (β cell depletion) [1]. Additionally, the Janus kinase (JAK) – signal transducers and activators of transcription (STAT) signaling pathway can be targeted with JAK inhibitors, members of an evolving class of agents referred to as targeted synthetic DMARDs (tsDMARDs) [1]. In this 2024 update of the Asia‐Pacific League of Associations for Rheumatology (APLAR) recommendations for treatment of RA, the term “targeted therapy” covers both bDMARDs and tsDMARDs and is denoted “b/tsDMARDs”.

Our original 2015 APLAR guideline summarized international clinical practice recommendations for RA published from January 2000 to December 2013 [5]. An updated APLAR guideline was published in 2018 to include newer literature, with a focus on the growing evidence for targeted therapies [6]. Due to the expanding range of approved targeted therapies and their growing use in the Asia‐Pacific (AP) region, a review and revision of this guidance is warranted.

The 2024 APLAR guideline update summarized herein was developed in accordance with the overarching principles summarized in Box 1. Pertinently, these augmented recommendations were developed with AP patient populations in mind and considered the optimization of medical outcomes and provision of effective solutions for resource‐limited settings in some countries within the AP region. In circumstances where these aims are incompatible, we have offered guidance for both situations. Importantly, we believe that clinicians should discuss overall therapeutic goals and appropriate treatment options with patients in a shared decision‐making process, considering all pertinent risk factors when prescribing therapy.

BOX 1 **Overarching Principles Guiding APLAR 2024 Update.**

- RA treatment should be initiated without delay upon diagnosis and adjusted based on a shared decision by both the patient and the clinician to maintain physical functioning and good quality of life through achieving sustained remission, or low disease activity when remission may not be an achievable target.
- Healthcare providers should strive to ensure access to the best possible treatment(s) for patients with RA.
- The choice of treatment is based on disease activity and risk assessment that includes prognostic factors and comorbidities.
- Routine assessment of disease activity is essential to achieving treatment goals. If the desired target is not achieved, treatment should be modified. Once the target is attained, it should be maintained. Tapering should be considered in patients who achieve stable remission.
- Rheumatologists should ideally be the principal healthcare providers for patients with RA.

## 2 Materials and Methods

In this 2024 update to the APLAR treatment guidelines for RA, Steering Committee members involved in developing the 2015 and 2018 guidelines were invited to form the Working Group for the update. During the first of three meetings, the Working Group re‐evaluated clinical questions that guided the literature search for the 2015 and 2018 APLAR recommendations. The Working Group reached agreement on topics for discussion that formed the basis for the literature search strategy for the 2024 update.

Following the first meeting, we searched MEDLINE via PubMed, EMBASE, and the Cochrane Library for relevant randomized controlled trials (RCTs), observational studies, guideline publications, and meta‐analyses. Although literature published from January 2018 to August 2023 (following the 2018 APLAR update) was prioritized, older literature was revisited where relevant to the topic. Articles were assigned for review by Working Group members, who evaluated the evidence reported according to the Grading of Recommendations, Assessment, Development and Evaluations (GRADE) approach, which classifies quality of evidence considering factors such as study limitations, inconsistency of results, imprecision, and reporting bias, and classifies recommendations as “strong” or “weak” depending on evidence quality and uncertainty of the balance of benefit versus risk, variability in values, and other factors [7].

In the second and third meetings, the Working Group presented and discussed published evidence to ascertain its relevance to the 2024 guideline update. Using the GRADE approach, the strength of a recommendation and quality of evidence (“very low,” “low,” “moderate,” and “high”) were assigned grades to yield a single overall grade (Table 1) [8]. The Working Group drafted recommendation statements that were then refined during the meeting and in subsequent correspondence as the discussion of evidence continued. Using a modified Delphi technique, the voting group rated their agreement with each recommendation on a five‐point Likert scale (5, strongly agree; 4, agree; 3, neither agree nor disagree; 2, disagree; 1 strongly disagree); agreement (i.e., “strongly agree” or “agree”) by 75% of all voting members was defined as the threshold for accepting a statement.

**TABLE 1:** Meaning of GRADE levels for quality of evidence.

GRADE	Quality	Meaning
A	High	We are very confident that the true effect lies close to that of the estimate of the effect
B	Moderate	We are moderately confident in the effect estimate The true effect is likely to be close to the estimate of the effect, but there is a possibility that it is substantially different
C	Low	Our confidence in the effect estimate is limited The true effect may be substantially different from the estimate of the effect.
D	Very low	We have very little confidence in the effect estimate The true effect is likely to be substantially different from the estimate of the effect

*Note:* Reproduced from Balshem et al. [8].

Following the third meeting, a voting group, comprising country representatives nominated by the national rheumatology associations within APLAR, was convened. After the meeting, online discussions were used to finalize the wording of each statement, and a further confirmatory vote was taken using the same Likert scale and agreement threshold as used in earlier rounds of evaluation.

## 3 Results

Fourteen statements achieved the threshold for consensus. Table 2 presents each statement with its level of agreement (percentage of experts responded “strongly agree” or “agree” during evaluation), overall GRADE, strength of recommendation (1, strong; 2, weak), and a discussion of the supporting evidence follows below.

**TABLE 2:** Summary of recommendations.

Statement	Agreement	GRADE	Strength
1. Assessing disease activity and safety before initiating therapy and monitoring during therapy, preferably every 1–3 months, is recommended	100%	A (High)	1 (Strong)
2. A validated and practical standardized measure of disease activity should be routinely performed to measure disease activity and to assess patients' response to treatment	100%	B (Moderate)	1 (Strong)
3. Methotrexate should be initiated at a dose of 7.5–15.0 mg/week and rapidly escalated to the maximum tolerated and effective dose as needed. In the case of methotrexate intolerance, other csDMARDs may be considered	95%	B (Moderate)	2 (Conditional)
4. Oral glucocorticoids may be considered for the control of active rheumatoid arthritis as a bridging therapy together with csDMARDs at a dose of less than 7.5 mg/day for prednisolone or equivalent and should be tapered and discontinued as soon as possible	95%	B (Moderate)	1 (Strong)
5. In patients who have an inadequate response to methotrexate monotherapy without poor prognostic factors, the addition of other csDMARDs such as sulfasalazine and/or hydroxychloroquine may be considered	100%	B (Moderate)	2 (Conditional)
6. A b/tsDMARD should be promptly considered without delay in patients with poor prognostic factors and inadequate response to csDMARDs, as well as in those with csDMARD intolerance	100%	A (High)	1 (Strong)
7. Patients who do not achieve remission or low disease activity with b/tsDMARD therapy are recommended to switch to another b/tsDMARD, preferably with a different mechanism of action	100%	B (Moderate)	1 (Strong)
8. b/tsDMARDs are most effective when combined with a csDMARD, particularly methotrexate. For patients intolerant to csDMARDs, an IL‐6 pathway inhibitor or a tsDMARD may be preferred for monotherapy. Additionally, some TNF inhibitors are approved for monotherapy and may be considered	100%	B (Moderate)	1 (Strong)
9. After achieving sustained remission for at least 6–12 months, tapering of cs/b/tsDMARDs may be considered following glucocorticoid withdrawal	100%	A (High)	1 (Strong)
10. cs/b/tsDMARDs may be tapered to the lowest effective dose	100	B (Moderate)	1 (Strong)
11. If vaccination is required during treatment, temporary discontinuation of methotrexate and delayed administration of rituximab for 1–2 weeks after vaccination may be considered if disease activity allows	95%	B (Moderate)	2 (Conditional)
12. Screening and management of tuberculosis, hepatitis B and hepatitis C infections are recommended before initiating any DMARD treatment, following national guidelines. Patients with active or chronic infections should be referred to the appropriate specialists	100%	A (High)	1 (Strong)
13. Surveillance is necessary for detecting malignancy and assessing the risk of cardiovascular disease	90%	B (Moderate)	2 (Conditional)
14. Patients with rheumatoid arthritis who are pregnant or planning pregnancy and require medical therapy should continue or switch to pregnancy compatible DMARDs to control disease activity	100%	B (Moderate)	2 (Conditional)

*Note:* Agreement is the percentage of respondents who replied “strongly agree” or “agree” during evaluation.

Abbreviations: b, biologic; cs, conventional synthetic; DMARD, disease‐modifying antirheumatic drug; IL‐6, interleukin 6; LoE, level of evidence; TNF, tumor necrosis factor; ts, targeted synthetic.

### 3.1 Monitoring

Statement 1 *Assessing disease activity and safety before initiating therapy and monitoring during therapy, preferably every 1–3 months, is recommended*.

**Agreement: 100%; GRADE: A (High); Strength: 1 (Strong)**

In alignment with other expert groups [5, 9, 10] we recommend that disease activity be monitored at 1 to 3‐monthly intervals. This is a slightly modified version of Statement 5 in the 2015 APLAR guidance that recommended 1–3 monthly monitoring for all patients with recently diagnosed RA or active disease [5]. For patients with higher disease activity, shorter monitoring intervals (e.g., monthly monitoring) are preferable.

Evidence supporting this recommendation is found in several key studies. A pooled analysis of SDAI and CDAI scores from clinical trials concluded that patients treated with bDMARDs and/or MTX who did not achieve a minor response (SDAI 50%) at 3 months were highly unlikely to achieve treatment targets by 6 months [11], showing the importance of early and frequent monitoring.

Additional evidence is found in a randomized study comparing two strategies: a conventional MTX treatment approach (clinic visits, assessments and dose adjustments every 3 months) and an intensive approach with monthly intervals [12]. More patients in the intensive group achieved a period of remission compared with the conventional group during the 2‐year study period (76 [50%] vs. 55 [37%], respectively; *p* = 0.03) [12].

Further evidence of the benefits of early and frequent monitoring is found in the BeSt Study. In this study, patients were randomized to four groups: sequential DMARD monotherapy, step‐up combination therapy, initial combination therapy with tapered high‐dose prednisone, or initial use of the TNF inhibitor infliximab, with all groups having treatment adjustments at 3‐monthly intervals [13]. Although patients in the initial combination and infliximab groups experienced earlier functional improvement than the other groups (*p* < 0.001), there was marked improvement in disease activity across all four groups [13]. Overall, 32% of patients achieved remission (DAS44 of < 1.6) at the end of the first year, which the investigators attributed to frequent monitoring and treatment adjustments [13].

Frequent monitoring may also help reduce the burden of RA on the healthcare system in the long‐term. In an extension study of a randomized trial comparing RA patients with “direct access” (reviews initiated at patient request with access to a rheumatologist within 10 working days and immediate access to advice from nurses) to usual practice (3‐ or 6‐monthly reviews), the direct access group had 38% fewer hospital appointments over 6 years [14].

In some areas of the AP region, geographical access to rheumatology centers may pose a challenge to frequent monitoring. However, the COVID‐19 pandemic has accelerated the adoption of telemedicine in rheumatology, and APLAR recommendations regarding its use have been published [15].

Statement 2 *A validated and practical standardized measure of disease activity should be routinely performed to measure disease activity and to assess patients' response to treatment*.

**Agreement: 100%; GRADE: B (Moderate); Strength: 1 (Strong)**

This is a reiteration of the recommendation in APLAR's 2015 guidelines (Statement 6), with a minor wording update to emphasize that validated disease measures should be used [5].

A diverse and growing evidence base supports the routine use of disease activity scores in clinical practice, and the selection of a particular score can be made based on the local resources (e.g., for biomarker testing), and healthcare professionals' (HCP) and patients' preferences. Developments in digital health tools allow increased disease monitoring to be achieved without increasing clinic visits. A study comparing standard monitoring during clinic visits and “connected monitoring,” using a smartphone and self‐assessed disease scores, found the latter could reduce the number of HCP visits versus conventional monitoring in patients initiating a new DMARD [16]. Patients in the conventional and connected monitoring groups had similar rates of low disease activity (Disease Activity Score using 28 joint counts; DAS28 ≤ 3.2, 62.8% vs. 59.1%, respectively) or remission (DAS28 < 2.6, 46.5% vs. 36.4%) at 6 months [16].

Evidence from randomized controlled trials (RCTs) also demonstrates the benefits of planned monitoring. In a randomized 24‐week study, scheduled measurement of DAS28 was associated with more DMARD adjustments and a higher proportion of patients achieving a DAS28 score of ≤ 3.2 versus standard care (31% vs. 16%, respectively; *p* = 0.028) [17]. Similarly, the randomized Tight Control of Rheumatoid Arthritis (TICORA) study showed that intensive management, which included monthly assessments of a validated disease activity score, led to greater improvements in disease activity, radiographic progression, physical function and quality of life compared with patients receiving routine care [18].

A limitation of some widely‐used disease scores (e.g., DAS28, simplified disease activity index [SDAI] and clinical disease activity index [CDAI]) is their non‐specificity or inclusion of subjective measures of disease activity [19]. The multi‐biomarker disease activity (MBDA) is an objective measure of disease activity for RA patients designed to complement these conventional measures [19], and a systematic review and meta‐analysis showed the MBDA correlates with many conventional scores and is a reliable prognostic marker of radiographic progression [19]. Additionally, patients' perceptions of their disease severity are important. A study examining multiple disease activity measures in relation to patients' perceptions of their own symptomatic status (patient acceptable symptom state; PASS) found that the CDAI demonstrated the best performance regarding PASS [20].

Overall, these data emphasize the fundamental role of regular disease activity measurement during RA therapy. The use of biomarker‐based disease activity scores such as MBDA may not be practical in resource‐limited settings. In these settings validated composite disease activity indices that do not need laboratory markers, e.g., the CDAI, can suffice [5]. Additionally, in resource‐limited settings with limited access to rheumatologists, rheumatology nurses can play a key role in patient education and monitoring. A randomized study of stable RA patients in Hong Kong found that nurse‐led consultation was non‐inferior to rheumatologist consultation and had high patient satisfaction [21].

### 3.2 Use of csDMARDs

Statement 3 *Methotrexate should be initiated at a dose of 7.5–15.0 mg/week and rapidly escalated to the maximum tolerated and effective dose as needed. In the case of methotrexate intolerance or contraindication, other csDMARDs may be considered*.

**Agreement: 95%; GRADE: B (Moderate); Strength: 2 (Conditional)**

Methotrexate remains the first line of therapy for treatment‐naïve patients in agreement with the APLAR 2015, APLAR 2018 and other guidelines [5, 6, 9, 22]. This statement merges statements 17 and 18 of the APLAR 2015 guidance which note MTX as an “anchor drug” [5]. Additionally this revised version is more detailed than a similar recommendation in Statement 1 of the 2018 update and now includes a suggested dose [6]. The utility of MTX monotherapy as an initial RA therapy is supported by a meta‐analysis of studies comparing MTX monotherapy to MTX‐containing DMARD combinations, that revealed that oral MTX monotherapy had a 40.5% response rate for achieving American College of Rheumatology 50% response (ACR50) in MTX‐naïve patients, (vs. 61.2% for MTX + SSZ + HCQ) [23]. The expected radiographic progression for patients on MTX monotherapy was 2.34 points over 1 year (below the minimal clinically important difference of 5 units on the Sharp‐van der Heijde scale), with a withdrawal rate due to AEs of 76 per 1000 patients within that time frame [23].

Evidence from systematic reviews supports the initiation of oral MTX at a dose of 15 mg/week and escalating to 25–30 mg/week (or the highest tolerated dose) [24, 25]. The efficacy and tolerability of MTX appear to be independent of the route of administration or whether the oral doses of MTX are split [26]. Furthermore, rapid dose escalation (5 mg every 2 weeks) from 15 to 25 mg/week is not associated with significant efficacy or tolerability differences compared with slower escalation (5 mg every 4 weeks), with the exception of more frequent gastrointestinal adverse events (AE) during the initial 8 weeks of rapid escalation (40% vs. 27%; *p* = 0.048) [27]. Parenteral administration is an option for patients with poor response or intolerance to oral MTX [24, 25, 26].

Statement 4 *Oral glucocorticoids may be considered for the control of active rheumatoid arthritis as a bridging therapy together with csDMARDs at a dose of less than 7.5 mg/day for prednisolone or equivalent and should be tapered and discontinued as soon as possible*.

**Agreement: 95%; GRADE: B (Moderate); Strength: 1 (Strong)**

This statement merges Statements 13, 14 and 15 of the 2015 APLAR guidance [5], and is in agreement with other expert groups [9, 28, 29, 30], we suggest oral glucocorticoids be considered as a bridging therapy in combination with a DMARD to manage disease. The recommendation against oral glucocorticoid monotherapy included in the 2015 APLAR recommendations has not been reiterated here [5], but is implicit in this statement. Glucocorticoids can delay both the onset and progression of joint damage [31]. Therefore, a short course of glucocorticoid therapy (i.e., 3–6 months) may be appropriate for managing disease flares in adults with recent onset or established disease. This approach can rapidly reduce inflammation while waiting for the effects of a newly initiated csDMARD regimen [31, 32]. However, a notable exception to this approach is that the 2021 ACR guidelines strongly recommend initiating csDMARDs without the use of longer‐term (≥ 3 months) glucocorticoids in DMARD‐naïve patients with moderate‐to‐high disease activity [22].

Different regimens and routes of administration can be employed for glucocorticoid therapy, but they should always be tapered and discontinued as soon as possible [9, 31]. The European Alliance of Associations for Rheumatology (EULAR) guidelines emphasize that csDMARDs and bDMARDs should be used to avoid chronic glucocorticoid therapy [9].

The efficacy of adding glucocorticoids to DMARDs is supported by multiple randomized studies [33, 34], with the GLORIA trial and CareRA being two notable examples [35]. The flexibility of glucocorticoid regimens is exemplified in CareRA, which included randomized groups representing all three COBRA (Combination therapy for early RA) regimens and a “tight step‐up” regimen [36]. The COBRA‐Slim regimen (MTX combined with glucocorticoid bridging) provided the best balance between efficacy and safety [36], but all regimens combining DMARDs with glucocorticoids proved effective for patients with early RA up to 2 years [36].

A post hoc analysis of phase 3 studies suggests that glucocorticoids should not be added to tofacitinib therapy due to a lack of efficacy [37], and emerging data from two small studies suggest that tofacitinib can be used as a steroid‐sparing agent, allowing glucocorticoids to be discontinued in up to 30% of patients [38, 39].

Long‐term use of glucocorticoids should be avoided due to their AE profiles, namely the risk of infections, fractures and cardiovascular (CV) events [9, 34, 40, 41]. The risk of infections associated with glucocorticoid use is influenced by both the dose and duration of use [34, 42], and is of particular relevance to rheumatologists in the AP region, given the high prevalence of tuberculosis and hepatitis in some countries [43, 44]. Although daily prednisone doses of ≤ 4 mg or with shorter cumulative doses and durations do not appear to be associated with increased CV risk, such risks are associated with daily doses ≥ 5 mg and increase with cumulative dose and duration of use [45]. Results from a multicenter, double‐blind, placebo‐controlled RCT compared the effects of high (60 mg/day) and low (10 mg/day) doses of prednisolone as short‐term bridging strategies in active early RA. This study showed no benefit from short‐term glucocorticoid bridging therapy on the progression of radiographic damage after 1 year at either dose [46]. A retrospective observational study that compared daily and alternate‐day dosing of adjunctive glucocorticoid treatment concluded that alternate‐day dosing is preferable to daily dosing to minimize AEs [47].

Statement 5 *In patients who have an inadequate response to methotrexate monotherapy without poor prognostic factors, the addition of other csDMARDs such as sulfasalazine and/or hydroxychloroquine may be considered*.

**Agreement: 100%; GRADE: B (Moderate); Strength: 2 (Conditional)**

The addition of another csDMARD to MTX, namely SSZ and/or HCQ, in patients with inadequate response to MTX monotherapy is an evolution of the principles of Statement 18 of the 2015 and Statement 2 of the 2018 APLAR guidelines, which recommend switching patients who cannot tolerate MTX to other csDMARDs and is consistent with guidance from other expert groups [5, 6, 9, 22].

This approach is supported by an RCT comparing the addition of glucocorticoids to MTX + HCQ against the addition of placebo [33]. Although the glucocorticoid group experienced a more rapid decrease in the DAS28‐ESR score, both groups achieved similar scores by month 12 [33]. Additionally, there was no statistically significant difference between the two groups, which were defined as a DAS28‐ESR < 2.6 (62.5% remission in the glucocorticoid group vs. 55% in the placebo group) [33]. Further support for intensifying MTX therapy with the addition of csDMARDs comes from a meta‐analysis of 158 RCTs that compared MTX monotherapy with MTX‐containing DMARD combinations [23]. For patients with an inadequate response to MTX, the addition of HCQ, SSZ or both resulted in probabilities of achieving an ACR50 response of 56.6%, 26.7% and 60.5%, respectively, compared with 12.7% for MTX alone [23]. Moreover, a change in radiographic progression over 1 year was 0.70 points for the combination of MTX + HCQ and SSZ compared with 3.35 points for MTX alone [23].

### 3.3 Use of Targeted Therapies

Statement 6 *A b/tsDMARD should be promptly considered without delay in patients with poor prognostic factors and inadequate response to csDMARDs, as well as in those with csDMARD intolerance*.

**Agreement: 100%; GRADE: A (High); Strength: 1 (Strong)**

This statement recapitulates Statement 7 of APLAR 2018 and Statement 26 of APLAR 2015, both of which recommend targeted b/tsDMARD initiation in patients with poor response or intolerance of csDMARDs and Statement 27 of APLAR 2015 which endorses “early” bDMARD use in patients with active disease and poor prognostic factors [5, 6]. Poor prognostic factors typically includes persistently high disease activity (after csDMARDs), autoantibody positivity, early presence of erosions, failure of ≥ 2 csDMARDs, among others [9, 48]. Initiating b/tsDMARD treatment in patients with an intolerance or poor response to csDMARD therapy, as defined by a treat‐to‐target goal, aligns with recommendations from other expert groups and may be preferable to triple csDMARD therapy [9, 22]. High‐quality evidence for this statement comes from two systematic reviews and a meta‐analysis that included only RCTs [49, 50].

The meta‐analysis by Wang et al. reviewed 20 RCTs evaluating JAK inhibitors (tofacitinib, *n* = 12, baricitinib *n* = 5 or upadacitinib *n* = 3) in patients with inadequate response to other DMARDs [49]. Compared with placebo, treatment with JAK inhibitors significantly increased the ACR20 response rate (relative risk [RR] 2.03; 95% confidence interval [CI] 1.87 to 2.20; *p* < 0.001) and resulted in lower Health Assessment Questionnaire‐Disability Index (HAQ‐DI) scores (mean difference 0.31; 95% CI 0.34 to 0.28; *p* < 0.001) [49]. Further support is provided by a network meta‐analysis by Weng et al. that included 88 RCTs of RA patients who had an inadequate response to csDMARDs [50]. Patients in this analysis received targeted therapy, either JAK inhibitors or bDMARDs [50]. All targeted therapies assessed were found to be significantly superior to placebo for ACR20 response rates (odds ratios [ORs] from 3.05 to 5.61), and HAQ‐DI scores (weighted mean differences from −0.34 to −0.21) [50].

Statement 7 *Patients who do not achieve remission or low disease activity with b/tsDMARD therapy are recommended to switch to another b/tsDMARD, preferably with a different mechanism of action*.

**Agreement: 100%; GRADE: B (Moderate); Strength: 1 (Strong)**

Switching from one targeted therapy to another with a different mechanism of action in patients who fail to respond is a concept endorsed in multiple guidelines [6, 9, 22, 28]. This statement is an evolution of Statement 33 of the 2015 APLAR guidance and Statement 12 of the APLAR 2018 guidance, but explicitly adds the preference for different mechanism of action [5, 6].

A systematic review informing the 2019 EULAR update reinforces that in patients with an inadequate response to TNF inhibitors or other bDMARDs, both tsDMARDs and bDMARDs (from the same or different class) generally result in a similar efficacy [51]. Some studies indicate moderate improvements in efficacy from switching within the same class—referred to as “cycling.” For example, in the EXXELERATE study, patients with an inadequate response to MTX were randomized to certolizumab pegol or adalimumab, both in addition to MTX [52]. Patients had the option to cycle between these TNF inhibitors at week 12 if they had an inadequate response to the first treatment [52]. Among those who did so, 58% of those cycled to certolizumab pegol and 62% of patients cycled to adalimumab, showed a DAS28 ESR reduction of ≥ 1.2 points at 12 weeks [52]. Another positive example of cycling is EXTEND, an extension of the ASCERTAIN study, which evaluated switching from intravenous (IV) tocilizumab to subcutaneous (SC) sarilumab (both anti‐IL‐6 agents) [53]. Patients who cycled in this study maintained clinical efficacy to 96 weeks, and no new safety concerns were noted [53].

A broader evidence base, comprising multiple RCTs, supports the benefits of switching between different classes of targeted therapy. In an RCT involving 300 patients with inadequate response to TNF inhibitors, a higher proportion of patients achieved a good or moderate EULAR response when randomized to a non‐TNF‐targeted bDMARD compared with those who cycled to a second anti‐TNF agent (odds ratio [OR] 2.06; 95% CI 1.27 to 3.37; *p* = 0.004) [54] Similarly, the SARIL‐RA‐TARGET RCT confirmed that that switching to sarilumab after inadequate response to TNF inhibitors improved the signs and symptoms of RA and physical function [55]. In SELECT‐COMPARE, switching from upadacitinib to adalimumab or vice versa helped patients achieve meaningful clinical responses after inadequate response to their first alternative [56]. In SELECT‐CHOICE, patients with RA and inadequate response to bDMARDs were randomized to upadacitinib or abatacept, and greater improvements in 12‐week DAS28‐CRP at week 12 were seen with upadacitinib (−2.52 vs. −2.00), respectively (difference, −0.52; 95% CI −0.69 to −0.35; *p* < 0.001 [superiority]) [57].

High‐quality evidence for the timing of switching is lacking, but other guideline publications have suggested 6 months as an appropriate time point [6, 28]. Similarly, evidence on whether patients with inadequate response to JAK inhibitors should be switched within this class or to other classes of agents is limited to observational studies [58, 59, 60].

Statement 8 *b/tsDMARDs are most effective when combined with a csDMARD, particularly methotrexate. For patients intolerant to csDMARDs, an IL‐6 pathway inhibitor or a tsDMARD may be preferred for monotherapy. Additionally, some TNF inhibitors are approved for monotherapy and may be considered*.

**Agreement: 100%; GRADE: B (Moderate); Strength: 1 (Strong)**

This statement is an evolution of Statement 8 of APLAR 2018, which noted that all targeted therapies are equally effective when combined with MTX or csDMARDs [6], and Statement 31 of APLAR 2015 that noted while bDMARD monotherapy is an option, bDMARDs are most effective when combined with MTX [5]. It is also similar to Statement 9 of the EULAR 2022 update which suggests the use of IL‐6 inhibitors and tsDMARD in patients who cannot use csDMARDs as a comedication [9]. Core evidence for this guidance is found in three meta‐analyses and five RCTs. Two of the meta‐analyses assessed the effectiveness of bDMARDs or tofacitinib combined with csDMARDs (mostly MTX), with ACR50 as the primary outcome for both [61, 62]. The analysis of Donahue et al. included 22 studies that compared TNF inhibitors +MTX or non‐TNF‐directed biologics + MTX with biologic monotherapy in patients with early RA [61]. Combination therapy—either adalimumab +MTX or etanercept + MTX—consistently outperformed biologic monotherapy. Relative risks for ACR50 response were 1.52 (95% CI 1.28–1.80) for adalimumab + MTX versus adalimumab monotherapy and 1.57 (95% CI 1.23–2.02) for etanercept versus etanercept monotherapy [61]. Similar results were seen in comparisons of abatacept + MTX with abatacept monotherapy and tocilizumab + MTX with tocilizumab monotherapy [61].

In the second meta‐analysis, the patient population had been treated with MTX or other DMARDs, and interventions included all bDMARDs + MTX/csDMARD vs. comparator (MTX/csDMARD/placebo) [62]. In this analysis, bDMARDs + MTX/csDMARD was associated with a significant and meaningful ACR50 improvement versus the comparator (RR 2.71; 95% CI 2.36 to 3.10) [62]. In a network meta‐analysis of phase 3 RCTs that compared adalimumab to other targeted therapies in combination with MTX [63], surface under the cumulative ranking curve (SUCRA) analysis found that upadacitinib + MTX, baricitinib + MTX, and tofacitinib + MTX rated highest for the likelihood of ACR50 response [63].

Additional evidence for anti‐IL‐6 agents as monotherapy or in combination with csDMARDs can be found in four RCTs conducted in patients with inadequate response to MTX or TNF inhibitors [55, 64, 65, 66]. These studies included SARIL‐RA‐TARGET, which demonstrated that sarilumab + csDMARDs leads to a higher achievement of ACR20 compared with csDMARDs alone [55]; KAKEHASI, which reported improved ACR20 achievement with sarilumab than with placebo [66]; SURPRISE, which compared adding tocilizumab to MTX with switching from MTX to tocilizumab [67]; and CREDO 1 and CREDO 3 studies showed better ACR20 responses with olokizumab compared with MTX [64, 65].

Statement 9 *After achieving sustained remission for at least 6–12 months, tapering of cs/b/tsDMARDs may be considered following glucocorticoid withdrawal*.

**Agreement: 100%; GRADE: A (High); Strength: 1 (Strong)**

Statement 10 *cs/b/tsDMARDs may be tapered to the lowest effective dose*.

**Agreement: 100%; GRADE B (Moderate); Strength: 1 (Strong)**

This statement is a broader recommendation for tapering than those included in previous APLAR recommendations. Statement 10 of APLAR 2015 recommended gradual tapering of csDMARDs after 6–12 months of remission after discontinuation of non‐steroidal anti‐inflammatory drugs, corticosteroids and bDMARDs, and dose reduction or tapering of targeted therapy after > 12 months of remission of disease is suggested in the 2015 and 2018 APLAR guidelines (Statements 34 and 13, respectively) [5, 6] The ACR and EULAR guidelines also recommend tapering of cs/b/tsDMARDs after 6 months of sustained remission (or low disease activity in the ACR guidance) [9, 22]. The decision to taper should be based on a shared decision with the patient, following a clear discussion of the risk of relapse. In the systematic review informing the 2022 EULAR update, a review of 47 studies of cs/b/tsDMARDs concluded that tapering was feasible for some patients, provided that they remain in remission or low disease activity [68].

Several studies have evaluated dose reduction strategies in patients with RA. These include a single‐center trial that enrolled patients with DAS28 remission for ≥ 6 months on etanercept 50 mg once weekly [69]. Patients were randomized to continue weekly treatment (*n* = 34) or change to etanercept 50 mg every other week (EOW, *n* = 32) [69]. After 6 months, 76% and 59% of patients in the weekly and EOW groups, respectively, maintained disease control, a difference that was not statistically significant (*p* = 0.136) [69]. A similar approach has been demonstrated in a study of patients with active RA despite csDMARD therapy or a single bDMARD who received tocilizumab 162 mg once weekly as monotherapy or with the csDMARD combination during a 24‐week single‐arm phase [70]. Patients achieving remission (DAS28 ≤ 2.8) at weeks 20 and 24 were then randomized to tocilizumab 162 mg weekly or Q2W for 24 weeks [70]. In the single‐arm phase, 45% (*n*/*N*; 179/401) patients achieved remission, and of those randomized at week 24, 90% of patients in the weekly tocilizumab group and 73% in the Q2W group remained in remission at week 48 (*p* = 0.004) [70]. Although this difference was statistically significant, it demonstrated that some patients were able to sustain remission on a less frequent dose of tocilizumab [70]. Therefore, tocilizumab Q2W dosing may represent an option for selected patients who find weekly dosing impractical or unaffordable.

Evidence also supports dose reduction of rituximab. In the REDO RCT, patients with RA and good response to rituximab at standard doses were randomized to one dose of either 1000 mg (*n* = 29), 500 mg (*n* = 58) or 200 mg (*n* = 55) [71]. The per‐protocol analysis found that the 500 mg dose was non‐inferior to 100 mg at 3 months but not at 6 months, and intention‐to‐treat analysis at 6 months found that both 500 mg and 200 mg rituximab were non‐inferior to 1000 mg [71]. The investigators concluded that a strategy of one ultra‐low dose of rituximab with additional DMARDs in case of flare may be feasible in clinical practice.

For adalimumab, the phase 4 PREDICTRA study, in which patients were randomized from adalimumab Q2W to adalimumab tapering (Q3W, *n* = 102), or the withdrawal arms (*n* = 20) [72]. Disease flares were experienced by 36% of patients in the taper group compared with 45% in the withdrawal group, and rescue adalimumab could restore disease control in some, but not all cases [72]. A pooled analysis of two studies evaluating down‐titration and discontinuation strategies for anti‐TNF bDMARDs concluded that discontinuation slightly increased the mean DAS28 score after 28 to 52 weeks (mean difference 0.96; 95% CI 0.67 to 1.25), and in an analysis of 6 RCTs, the RR of persistent remission was between 0.16 and 0.77 [73].

In general, evidence suggests that a gradual tapering of the dose is a better approach than discontinuation in patients who are in remission. A meta‐analysis of studies evaluating tapering of csDMARDs (*n* = 4 studies) or TNF inhibitor bDMARDs (*n* = 15) concluded that more than one‐third of patients may taper or stop treatment without experiencing a disease flare within the first year [74]. Notably, dose reduction of TNF inhibitor bDMARDs was associated with lower flare rates than discontinuation [74].

### 3.4 Vaccination and Screening for Infections, Cardiovascular and Malignancy Risks

Statement 11 *If vaccination is required during treatment, temporary discontinuation of methotrexate and delayed administration of rituximab for 1–2 weeks after vaccination may be considered if disease activity allows*.

**Agreement: 95%; GRADE: B (Moderate); Strength: 2 (Conditional)**

Previous APLAR consensus statements have not considered the temporary suspension of therapies to permit vaccination. Statement 11 is similar to guidance from the ACR, which recommends holding MTX therapy for 1–2 weeks after influenza vaccination if disease activity allows [75]. Rituximab should ideally be administered at least 2 weeks after the influenza vaccine of other non‐live vaccines.

This approach has demonstrated its utility with COVID‐19 and influenza vaccines [76, 77, 78, 79]. For example, in a randomized multicenter study in the UK, patients randomized to a 2‐week suspension of MTX (*n* = 191) immediately prior to the administration of a SARS‐CoV‐2 booster vaccine showed a significantly higher antibody titer than those randomized to continuation of MTX (*n* = 192; Geometric mean ratio of antibody titer: 2.08; 95% CI 1.59 to 2.70; *p* < 0.0001) [76]. Similarly, a single‐center randomized study in Brazil found that anti‐SARS‐CoV‐2 S1/S2 IgG seroconversion rates were higher in RA patients who suspended MTX for 2 weeks (*n* = 37) after each vaccine dose compared with those who continued MTX (*n* = 55, 78.4% vs. 54.5%; *p* = 0.019) [77]. A randomized multicenter study has also demonstrated the effectiveness of a 2‐week hold of MTX for improving vaccine responsiveness in RA patients receiving the seasonal influenza vaccine. Patients in the MTX‐hold group (*n* = 160) achieved a higher rate of satisfactory vaccine response (assessed by increase in hemagglutination inhibition antibody titer at 4 weeks after vaccination) than those in the MTX continuation group (*n* = 156, 75.5% vs. 54.5%; *p* < 0.001) [78]. A subsequent randomized multicenter study by the same investigators found that a 1‐week suspension of MTX after vaccination was non‐inferior to a 2‐week suspension for vaccine response as measured by hemagglutination assay (*N* = 178; 68.9% versus 75.0% with satisfactory response, respectively; *p* = 0.364) [79].

The administration of vaccines before rituximab treatment is supported by a study in Taiwan that found, compared with RA patients treated only with csDMARDs (*n* = 86), those receiving rituximab or abatacept (*n* = 142) exhibited significantly lower immune response to an mRNA SARS‐CoV‐2 vaccine (*p* = 0.008 and *p* = 0.035, respectively). This underscores the importance of completing vaccination prior to the initiation of rituximab to optimize immune response [80].

Statement 12 *Screening and management of tuberculosis, hepatitis B and hepatitis C infections are recommended before initiating any DMARD treatment, following national guidelines. Patients with active or chronic infections should be referred to the appropriate specialists*.

**Agreement: 100%; GRADE: A (High); Strength: 1 (Strong)**

Screening and management of tuberculosis (TB), hepatitis B virus (HBV) and hepatitis C virus (HCV) infections prior to initiation of DMARD treatment are endorsed in multiple guidelines including APLAR 2015 (Statements 35–37) and 2018 (Statement 5) [5, 6, 81].

Minimizing the risk of infections is a key consideration when using any DMARDs, and the evidence base for infection risk in patients treated with targeted therapy is expanding. A meta‐analysis of 39 RCTs found that treatment of RA with bDMARDs, particularly TNF inhibitors, significantly increased the risk of TB compared to non‐biologics (OR 3.86; 95% CI 2.36 to 6.32; *p* < 0.001) [82]. Trends towards increased risk with IL‐6 inhibitors and JAK inhibitors were also noted but were not statistically significant [82]. Similarly, a nationwide cohort study in Taiwan reported a 1‐year incidence rate of TB of 1.513% among RA patients initiating TNF inhibitor therapy, highlighting a significantly increased risk compared to patients treated with non‐TNF inhibitors (adjusted hazard ratio [HR] 7.19; 95% CI 4.18 to 12.34) [83]. Cohort studies on TB risk in RA patients in South Africa and Brazil, showing increased risks of TB infections in patients treated with biologics, reinforce these findings [84, 85].

Data from cohort studies conducted in Taiwan and Korea provide additional insights into infection risk with TNF inhibitors or JAK inhibitors. In a nationwide Korean cohort, infliximab was associated with a higher risk of developing TB compared to etanercept, particularly in patients without evidence of latent TB infection [86]. Data on the risk of infection with JAK inhibitor use compared with bDMARDs is mixed. A cohort study from Korea found a significantly lower risk of active TB in JAK inhibitor users compared with bDMARD users, indicating potential differences in TB risk among targeted therapy classes [87]. Conversely, a different Korean cohort study compared infection risks in RA patients treated with JAK inhibitors or TNF inhibitors and found a higher incidence of opportunistic infections, including TB, in JAK inhibitor users [88].

The risk of HBV infection has also been assessed in this setting. A meta‐analysis of 26 clinical and observational studies found an estimated HBV reactivation rate of 2.0% (95% CI 0.01 to 0.04) in patients treated with biologics or JAK inhibitors [89]. For HCV reactivation risk data are more reassuring. A retrospective study of 1548 patients with rheumatic diseases (35.3% with RA) treated with bDMARDs and csDMARDs reported that 25.5% tested positive for anti‐HCV antibodies, but no hepatitis C reactivation was observed during biologic therapy [90]. Nonetheless, HCV screening is essential before initiation of DMARD treatment.

Overall, these studies underscore the importance of systematic screening patients for TB, HBV and HCV infection prior to DMARD therapy. This is essential in the AP region where many countries have high endemicity [5, 6]. Patients with these infections should be referred to specialists for appropriate management to ensure their eligibility for treatment. More detailed discussion and guidance for screening and vaccination for infectious diseases including TB, HBV, HCV and herpes zoster in candidates for b/tsDMARD therapy can be found in the 2015 and 2018 updates to the APLAR guidelines [5, 6]. Additionally, physicians should refer to national vaccine recommendations for further guidance on this topic.

Statement 13 *Surveillance is necessary for detecting malignancy and assessing the risk of cardiovascular disease*.

**Agreement: 90%; GRADE: B (Moderate); Strength: 2 (Conditional)**

This statement is an evolution of our 2018 guidance to use targeted therapy with caution in patients with a history of solid tumors [6]. Reassuring data on these risks are found in a systematic review/meta‐analysis of 20 RCTs in RA patients that assessed the safety of tofacitinib, baricitinib and upadacitinib, including trials of monotherapy and combination therapy [49]. Venous thromboembolic events were considered in three studies using only upadacitinib, and no increase in risk was detected [49]. Malignancies were considered in seven studies, and the overall incidence of serious malignancy was similar to placebo (RR 1.68; 95% CI 0.57 to 4.95; *p* = 0.34) [49]. This is in contrast to an analysis of the ORAL Surveillance RCT, in which 4362 patients with RA and ≥ 1 CV risk factor were randomized to receive tofacitinib 5 mg or 10 mg twice daily or TNF inhibitors [91]. Incidence rates for malignancies excluding non‐melanoma skin cancer were higher with tofacitinib (individual doses and combined) than with TNF inhibitors (HR 1.48; 95% CI 1.04 to 2.09); this did not meet non‐inferiority criteria (HR 95% CI upper limit < 1.8) for combined tofacitinib doses versus TNFi [91]. Furthermore, malignancy risk was highest in patients with a history of atherosclerotic CV disease risk factors, suggesting there may be shared risk factors for CV risks and cancer [91].

Screening of patients may help minimize risks associated with targeted therapy [92]. An analysis from an observational cohort study conducted in Japan found that computed tomography (CT) screening for malignancies before initiating targeted therapy was more effective than standard screening [92]. Among 2193 patients who underwent CT scans prior to b/ts/DMARD initiation, 33 patients (1.5%) were diagnosed with malignancy. This approach enabled earlier detection and treatment of malignancy compared with standard screening, ultimately improving the safety of targeted therapy [92].

Based on these studies we recommend that prior to the initiation of targeted therapy, patients should be thoroughly assessed for cardiovascular and malignancy risk factors. This is particularly relevant in the AP region where resources are limited in many countries. This should include a detailed clinical and family history, physical examination, blood tests and chest x‐ray, with any abnormalities noted.

### 3.5 Use of DMARDs During Pregnancy

Statement 14 *Patients with rheumatoid arthritis who are pregnant or planning pregnancy and require medical therapy should continue or switch to pregnancy compatible DMARDs to control disease activity*.

**Agreement: 100%; GRADE: B (Moderate); Strength: 2 (Conditional)**

Statement 16 of the 2018 APLAR update notes that TNF inhibitors (preferably etanercept or certolizumab) may be continued during pregnancy in patients whose disease cannot otherwise be controlled [6]. This updated statement is informed by a meta‐analysis of 39 studies showed that an increased risk of preterm births (OR 1.45; 95% CI 1.16 to 1.82) and infections in newborns (OR 1.12; 95% CI 1.00 to 1.27) for women treated with TNF inhibitors for immune mediated inflammatory diseases, including inflammatory bowel disease (*n* = 21 studies), RA (*n* = 5), and psoriasis (*n* = 1) [93]. No significant differences were seen for caesarian sections, miscarriage, low birth weight, newborns small for gestational age and congenital malformations [93]. The binding affinity of an immunoglobulin G‐based biologic to the neonatal Fc receptor (FcRn) is an important consideration as biologics are transferred across the placenta via the same mechanisms as maternal antibodies [94]. This binding affinity is highest for monoclonal IgG1 antibodies (infliximab, adalimumab, golimumab, rituximab), low for Fc fusion proteins (etanercept and abatacept), and insignificant for pegylated Fc‐free molecules (e.g., certolizumab) [94].

The 2024 update from EULAR on the use of antirheumatic drugs in pregnancy notes that TNF inhibitors can be used throughout pregnancy [94]. Non‐TNFi bDMARDs including abatacept, anakinra, belimumab, canakinumab, ixekizumab, rituximab, sarilumab, secukinumab, tocilizumab, and ustekinumab may be used if needed as current evidence suggests they do not increase the rate of adverse pregnancy outcomes [94].

## 4 Discussion

This updated APLAR guideline intends to guide clinicians when choosing among the growing range of targeted therapies in clinical use in the AP region. The number of recommendations herein is decreased relative to the 2015 and 2018 APLAR updates [5, 6], as this report intends to complement rather than replace these earlier recommendations, and some statements have evolved due to expansion of the evidence base. The updated recommendation on baseline disease activity measurement before initiating any therapy has a clearer emphasis in this update, and we now include a precise dose of MTX in the statements, which was lacking in 2015 and 2018. In 2024, oral glucocorticoids are considered a bridging therapy and a dose and duration has been recommended (< 7.5 mg, for 3–6 months), whereas in 2015 the recommended dose was ≤ 7.5 mg and duration was up to 6 months [5]. In this update, tapering of any DMARD may be considered (following glucocorticoid withdrawal) after achieving sustained remission for at least 6–12 months, whereas in 2015 and 2018, the required duration for remission was 12 months [5, 6]. We also note in this update that csDMARDs may be tapered to the lowest effective dose, a topic not addressed in the 2015 and 2018 updates.

In the 2015 and 2018 update, initial combination csDMARDs were recommended for patients with moderate to high disease activity and poor prognostic factors [5, 6], whereas, whereas in 2024 this is recommended for only those without poor prognostic factors following inadequate response to MTX monotherapy. Our recommendations around the use of targeted therapies in patients with malignancy and CV risk have also evolved since the prior updates.

Although a substantial volume of new evidence has been published on therapy for RA in recent years, many questions remain. For example, head‐to‐head trials for many agents are unavailable, and although network meta‐analyses provide some data, they must be interpreted with caution. Due to the cost of prospective randomized studies, other means of comparing agents must be considered, such as registries of patients treated with targeted therapies. The ANSWER cohort in Kansai, Japan is an example of this approach in the AP region [95].

Despite the growing range of targeted therapies, some patients remain refractory to treatment, and the growing range of therapeutic options may lead to the problem of “clinical inertia” where patients undergo disease progression while they switch between multiple targeted therapy options before identifying one that is effective. Studies that identify biomarkers or other predictors of response or refractoriness may help reduce this problem and may be of particular value in parts of the APAC region where some patients must self‐fund therapy. There is also a need for additional safety data for all classes of DMARDs, particularly in special settings such as pregnancy.

The cost of newer agents such as bDMARDs and tsDMARDs remains a barrier in many parts of the AP region, where reimbursement is limited and many patients must self‐fund these therapies. It is crucial that physicians can identify patients most likely to benefit from these therapies and optimize their use to ensure care is cost‐effective. Data on generic csDMARDs and biosimilar DMARDs may help reduce cost barriers.

## 5 Conclusions

This update to the APLAR guidelines revisited older evidence for recommendations and reviewed newer evidence, particularly for topics concerning targeted therapy. As always, these recommendations are general in nature and should always be individualized according to the judgment of the treating physician.

## Author Contributions

Conception and design: Kunihiro Yamaoka, Zhanguo Li, Paul Bird, Manisha Bhochhibhoya, Yuko Kaneko, Chin Teck Ng, Keshav Raj Sigdel, Evan Vista, Chak Sing Lau. Acquisition of data: Khin Thin Zar Myo Aung, Manisha Bhochhibhoya, Minhaj Rahim Choudhury, Tsolmon Darisuren, Rudy Hidayat, Nguyen Van Hung, Wanruchada Katchamart, Babur Salim, Chak Sing Lau. Data analysis and interpretation: All authors. Manuscript drafting: Paul Bird, Minhaj Rahim Choudhury, Nguyen Van Hung, Wanruchada Katchamart, Chin Teck Ng, Shereen Ch'ng Suyin, Evan Vista, Chak Sing Lau. Manuscript revision for important intellectual content: All authors. All authors reviewed and approved the final version of the manuscript to be published. All authors agreed to be accountable for all aspects of the work, which includes ensuring that questions related to the accuracy or integrity of any part of the work are appropriately investigated and resolved, and being able to identify the contributions of each co‐author and ensure the integrity of their contributions.

## Funding

This work was supported by GlaxoSmithKline.

## Ethics Statement

The authors have nothing to report.

## Conflicts of Interest

Kunihiro Yamaoka: Honoraria: Pfizer, Chugai, Takeda, Astellas, AbbVie, Bristol‐Myers Squibb, Mitsubishi Tanabe, GlaxoSmithKline, Lilly, Janssen, Eisai, Teijin, Shionogi, Taisho, Actelion., Asahi Kasei, Ono Pharmaceutical, Nippon Shinyaku, Otsuka Pharmaceutical, Gilead Sciences, Daiichi Sankyo, Japan Tobacco, Boehringer Ingelheim, MSD, Hisamitsu Pharmaceutical, Sanofi, Ayumi Pharmaceutical, Nippon Kayaku, AstraZeneca, UCB, Nippon Medac, AMGEN; Consulting fees: Asahi Kasei, AbbVie, Gilead Sciences, Pfizer, Astellas, Lilly, Japan Tobacco; Grants or funds: Takeda, Pfizer, Astellas, Daiichi Sankyo, Lilly, Teijin, MSD; Patents, royalties and other disclosures: Patent jointly held with DNA Chip Research Inc. (no royalties received). Paul Bird: Advisory council or committee: Pfizer, UCB, Eli‐Lilly, Novartis, Abbvie; Consulting fees: Pfizer, UCB, Eli‐Lilly, Novartis, Abbvie. Yuko Kaneko: Honoraria: AbbVie, Asahi Kasei, Astellas, Boehringer Ingelheim, Bristol‐Myers Squib, Chugai, Daiichi Sankyo, Eisai, Lilly, Gilead, Janssen, Mitsubishi Pharma, Novartis, Pfizer, Taisho, UCB; Consulting fees: AbbVie, Asahi Kasei, Astellas, Boehringer Ingelheim, Bristol‐Myers Squib, Chugai, Lilly; Grants or funds: Asahi Kasei, Ayumi, Boehringer Ingelheim, Chugai, Eisai, Lilly, Gilead, Tanabe Pharma, Pfizer. Tsutomu Takeuchi: Honoraria: AbbVie, Chugai, Lilly, Eisai, Gilead Sciences, Pfizer, Taisho; Consulting fees: AbbVie, Lilly, Gilead Sciences, Tanabe, Taisho. The remaining authors declare no conflicts of interest.

## Data Availability

This review draws solely on previously published sources. No new datasets were generated or discussed, and all referenced materials have been disclosed in the bibliography.
